# The role of whole-genome sequencing for guiding systemic therapy in patients with soft tissue sarcoma

**DOI:** 10.1016/j.esmoop.2025.105287

**Published:** 2025-06-11

**Authors:** P. van der Laan, W.J. van Houdt, H. van Boven, P. Snaebjornsson, L.J.W. Bosch, K. Monkhorst, Y.M. Schrage, L. Heimans, J.M. Kerst, N. Steeghs, W.T.A. van der Graaf

**Affiliations:** 1 Department of Surgical Oncology, Netherlands Cancer Institute, Amsterdam, the Netherlands; 2 Department of Medical Oncology, Netherlands Cancer Institute, Amsterdam, the Netherlands; 3 Department of Pathology, Netherlands Cancer Institute, Amsterdam, the Netherlands; 4 Faculty of Medicine, University of Iceland, Reykjavik, Iceland; 5 Department of Medical Oncology, Erasmus MC Cancer Institute, Rotterdam, the Netherlands

**Keywords:** soft tissue sarcoma, whole-genome sequencing, biomarkers, personalized medicine

## Abstract

**Background:**

The analysis of tumor DNA by whole-genome sequencing (WGS) is increasingly utilized for the identification of clinically relevant DNA aberrations. We aim to assess whether WGS improves the guidance of individualized systemic therapy in soft tissue sarcoma (STS).

**Patients and methods:**

WGS results of STS patients were retrieved from electronic patient records and pathology reports from clinical studies and routine diagnostics. WGS was carried out in primary STS with an unfavorable prognosis, metastatic STS, or STS where broad molecular diagnostics were deemed necessary. Actionable targets were defined as genomic alterations identified by WGS for which specific systemic therapy was indicated in routine care or clinical trials available at time of the WGS analysis.

**Results:**

WGS was carried out on 161 STS patients. The most common histological subtypes were leiomyosarcoma (22%), undifferentiated pleomorphic sarcoma/sarcoma not otherwise specified (17%) and dedifferentiated liposarcoma (14%). The majority of WGS analyses were requested at the time of recurrence or metastasis (41%), At least one actionable target was found by WGS in 74 (46%) of patients. Actionable targets were more frequently seen for complex genome sarcomas compared with simple genome sarcomas (50% versus 28%). Eventually, 23 patients (14%) received matched experimental therapy based on their WGS results. Non-availability of WGS directed treatment or lack of clinical necessity for systemic therapy (*n* = 17) and rapid disease progression causing poor performance score (*n* = 10) were the main reasons to not start WGS-informed therapy.

**Conclusion:**

WGS identified actionable targets in 46% of STS cases, leading to WGS-informed therapy in 14% of patients. Improving the timing of the WGS request and a more appropriate patient selection upfront could increase this relatively low percentage. Complex genome sarcomas seem to be the STS group for which WGS is most likely to add value by opening the way to tumor-agnostic therapies.

## Highlights

• WGS results of 161 STS patients were assessed to determine the value of WGS in guiding individualized systemic therapy.
• In 46% of STS cases, actionable targets were identified using WGS, leading to WGS-informed therapy in 14% of patients.
• Complex genome sarcomas seem to be an STS subgroup that benefit most from target identification by WGS.
• In some STS subtypes there is more benefit from using WGS than in others.
• The optimal timing of WGS for therapeutic reasons depends on treatment availability and patients’ condition.

## Introduction

Soft tissue sarcomas (STS) encompass a group of rare malignant tumors that arise from mesenchymal tissue. STS arise in diverse anatomic locations and account for <1% of adult malignant tumors, with an annual incidence of up to five cases per 100 000 people.^1^ The enormous heterogeneity in STS is reflected by the existence of >100 different subtypes that are now recognized by the World Health Organization.^2^ Unfortunately, their biological and clinical complexity forms a barrier to improving clinical outcomes. Because every histological subtype has its own specific clinical and biological characteristics, optimal therapy requires a subtype-specific approach.

Sarcomas are primarily diagnosed based on tumor morphology and immunohistochemical profiles, often in conjunction with targeted molecular analysis. Accurate diagnosis is crucial to support clinical decision making. Some STS subtypes are characterized by a defined molecular alteration, such as an amplification of *MDM2* in dedifferentiated liposarcoma or *PAX3* or *PAX7* fusions with *FOXO1* in alveolar rhabdomyosarcoma.^3^^,^^4^ Molecular hallmarks continue to evolve as new molecular techniques have been introduced and new molecular alterations have been identified. This has led to a new tumor-agnostic therapy approach in oncology, where cancer therapy is focused on targeting specific genomic alterations rather than basing therapy on primary tumor location or histological tumor type.^5^ In the Dutch Drug Rediscovery Protocol study, 2500 patients with advanced or metastatic cancer were evaluated with the aim to administer therapy based on a molecular genomic event. Eventually, 1500 of them received therapy based on their genetic tumor profile.^6^ Clinical benefit rate was 33% for the total cohort.

Whole-genome sequencing (WGS) is proving to be a useful tool in clinical cancer care.^7^, ^8^, ^9^ WGS provides a comprehensive overview of the entire tumor DNA, in which all genomic alterations are mapped. A study that investigated WGS for the selection of personalized experimental therapy in a highly diverse cohort of tumor types reported actionable events in 84% of cases. A matched therapy was available for 69% of cases. In total, 35% eventually received biomarker-informed experimental therapy.^10^ Previous studies show that WGS during the diagnostic workup can refine diagnoses in STS.^11^^,^^12^ The usability of WGS to discover previously unrecognized genomic alterations in STS has been studied with data obtained from ‘The 100 000 Genomes Project’. From this project, launched in the UK in 2013 to apply WGS in the study of rare diseases, cancers and infections, the data of 350 sarcoma patients were analyzed.^12^^,^^13^ In 55% of cases, a genetic alteration with therapeutic or prognostic relevance was found. Unfortunately, the associated drugs were not accessible for most sarcoma patients. The results of this study underline the potential value of exploring the mutational landscape of STS, but at the same time reflect the challenges in developing new and effective targeted-therapy strategies in STS.

Here we share the results of a single center experience in the Netherlands on WGS analysis to evaluate whether WGS enhances the guidance of individualized systemic therapy in patients with STS. These results reveal commonly identified targets for systemic therapy, highlight STS subtypes that particularly benefit from WGS analysis through the identification of new therapeutic options, and help to determine the most optimal time in the disease course when WGS offers the greatest clinical value for guiding systemic treatment decisions.

## Methods

### Study population

Between February 2017 and February 2023, patients diagnosed with an STS at the Netherlands Cancer Institute were selected for WGS assessment. WGS analysis was carried out as part of the prospective Center for Personalized Cancer Treatment (CPCT-02) biopsy protocol (NCT01855477),^14^ or the prospective WGS Implementation in standard Diagnostics for Each cancer patient (WIDE) study,^15^ or as part of regular diagnostics (retrospective data). WGS was carried out on either a biopsy or resection specimen in the following settings: primary STS with an unfavorable prognosis, metastatic STS or STS where broad molecular diagnostics were deemed necessary. Pathological diagnosis was made by an STS expert pathologist. Patients with a gastrointestinal stromal tumor, benign soft tissue tumor or desmoid fibromatosis were excluded from the present study. STS were categorized into simple genome versus complex genome STS according to the pathologist. Simple genome STS were defined as chromosomally stable, containing a single mutating event. STS that were genetically more complex were classified as complex genome STS. Patient demographics and tumor characteristics were obtained from the electronic patient records. This study was approved by the Institutional Review Board of the Netherlands Cancer Institute (IRBd22-230).

### Sample collection and WGS analysis

Fresh tumor samples were obtained by regular tissue retrieval procedures and were submitted for WGS analysis to Hartwig Medical Foundation (Hartwig). Results were reported including all genomic alterations and potential biomarkers (somatic variants, copy number changes, structural variants), tumor mutational burden (TMB), tumor mutational load (TML), microsatellite status, homologous recombination deficiency (HRD) and their clinical implication for systemic therapy, i.e. whether the found alteration was actionable, or the found biomarker was predictive of response to a specific therapy. HRD was detected based on a classifier called ‘CHORD’, that could be indicative to therapy with poly ADP-ribose polymerase inhibitors as previously described.^16^ A description of the DNA sequencing and bioinformatics workflow has been published and is available on the Hartwig website.^17^^,^^18^ Handling of germline mutations for the different studies was as follows: CPCT-02 study: germline findings were not reported. WIDE study: after informed consent patients were given the option to report tumor-associated germline variants at time of study entry or time of WGS request. Pathogenic germline variants in a predefined set of genes with implications for tumor-directed therapy were either reported as inherited variants, along with an offer for routine clinical genetics counseling, or reported as variants present without reporting germline status.^19^ Routine diagnostics: All variants were reported as somatic variants. If the variant found together with the tumor type warranted further genetic counseling, patients were referred to the clinical genetics department. Patients were referred only if it could affect therapy decisions and clinical follow-up of patients and/or their families.^18^ WGS reports were initially made available directly to the treating physician. From mid-2018, WGS results were checked by a clinical molecular biologist before being disclosed to the treating physician. From 2019, WGS reports were analyzed and interpreted within the clinical context of the patient to decide the best possible treatment plan and/or clinical studies by a dedicated multidisciplinary molecular tumor board that consisted of clinical molecular biologists, pathologists, clinical geneticists and medical oncologists. An ‘actionable target’ was defined as a genomic alteration identified by WGS for which specific systemic therapy was indicated in routine care or clinical trials available in the Netherlands at time of the WGS analysis, using the iClusion database.^20^ Biomarkers that are known to predict therapy efficacy, such as TML for immune checkpoint inhibition, were also included in this definition.

### WGS-informed therapy

Whether and when to start WGS-informed therapy was decided by the treating physician. All drugs given based on WGS results were in the context of a clinical trial. Reasons to not start WGS-informed therapy even though an actionable target was found were obtained from medical records or were verified with the treating physician.

### Data analysis

Patient and tumor characteristics were summarized using descriptive statistics. Data were presented in frequency (%) of median (interquartile range: IQR). Results of WGS analysis and WGS-informed therapy were visualized using GraphPad Prism version 9.3.0.

#### Data availability

The data used in this study are available for scientific research upon request at Hartwig Medical Foundation through standardized procedures. The data access request procedure can be found at https://www.hartwigmedicalfoundation.nl/en/. Since patient-level genome-wide data are considered privacy sensitive, these data are exclusively available through an access-controlled procedure. This procedure is specifically designed to evaluate whether the applicable research aligns with both ethical and legal constraints in relevant legislation and the terms of the consent given by the patients. Clinical data were collected with permission from the Institutional Review Board of the Netherlands Cancer Institute (IRBd22-230).

## Results

### Baseline characteristics

A total of 201 patients who had a suspected STS and for which a WGS was requested during the study period were included. After exclusion of 40 patients where WGS analysis had failed or in cases where the tumor was reclassified as benign, gastrointestinal stromal tumor or non-STS tumor, 161 STS cases remained. Baseline characteristics are depicted in Table 1. The median patient age was 56 years (IQR 46-65 years). The most frequent STS subtypes were leiomyosarcoma (*n* = 36), followed by undifferentiated pleomorphic sarcoma/sarcoma not otherwise specified (UPS/sarcoma NOS, *n* = 28) and dedifferentiated liposarcoma (DDLPS, *n* = 22). Most patients had metastatic disease (*n* = 105) at the time of WGS.

**Table 1:** Baseline characteristics of patients with soft tissue sarcoma for which whole-genome sequencing was carried out

Patient characteristics	All patients (*n* = 161)	
	*n*	%
Median age at diagnosis, years (IQR)	56 (46-65)	
Sex		
Male	84	52
Female	77	48
FNCLCC grade		
1	12	7
2	54	34
3	48	30
Not available or applicable	47	29
Histological tumor type		
Leiomyosarcoma	36	22
UPS/sarcoma NOS	28	17
Dedifferentiated liposarcoma	22	14
Other	75	47
Disease stage at WGS		
Localized	34	21
Locally advanced	22	14
Metastatic	105	65

FNCLCC, Fédération Nationale des Centres de Lutte Contre le Cancer; IQR, interquartile range; Sarcoma NOS, sarcoma not otherwise specified; UPS, undifferentiated pleomorphic sarcoma; WGS, whole-genome sequencing.

Table 2 summarizes prior therapies received by patients with metastatic disease. Most patients had a history of surgery (*n* = 69). A total of 49 patients with metastatic disease had already received one or more lines of systemic therapy when WGS was requested.

**Table 2:** Therapy received by patients with metastatic soft tissue sarcoma before WGS

Therapy before WGS	Metastatic patients (*n* = 105)	
	*n*	%
Locoregional treatment		
Surgery	69	66
Radiotherapy	48	46
Systemic therapy	49	47
None	16	15
Number of lines of systemic therapy		
1	26	25
2	15	14
≥3	8	8

### Timing of WGS request and WGS requests across years

**Figure 1:**
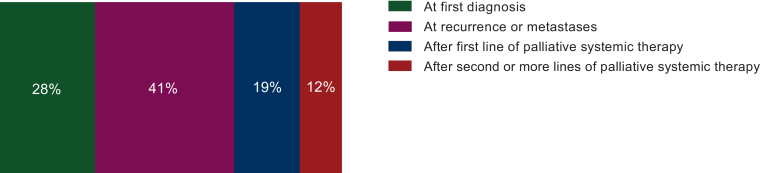
Timing of WGS request during disease course.

For all samples that underwent sequencing, the timing at which the WGS analysis was requested by the treating physician was recorded (Figure 1). For 28% of cases WGS analysis was carried out at first diagnosis. However, most WGS analyses were requested at the time of recurrence or metastasis [*n* = 66, (41%)], or later during the disease course: in 50 patients this was after at least one line of systemic therapy and in 8 patients after more than three lines of therapy. The number of WGS requests for STS patients has varied over the years, with a peak of 52 requests in 2020 during the WIDE study . Overall, there has been a increase in WGS requests for STS patients, rising from 8 in 2017 to 47 in 2022.

### Identification of actionable targets by WGS

**Figure 2:**
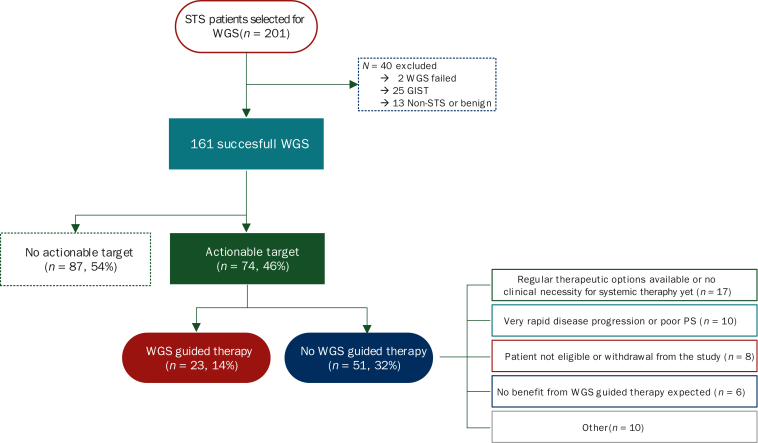
**Flowchart of WGS*-*analyzed patient samples and their actionability.** GIST, gastrointestinal stromal tumor; PS, performance status; STS, soft tissue sarcoma; WGS, whole-genome sequencing.

**Supplementary Figure S1:**
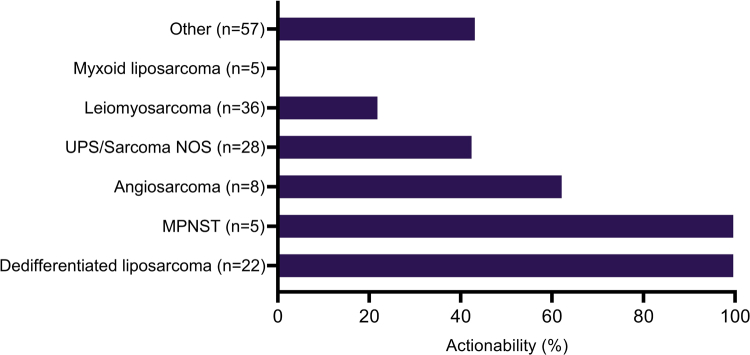


**Supplementary Figure S2:**
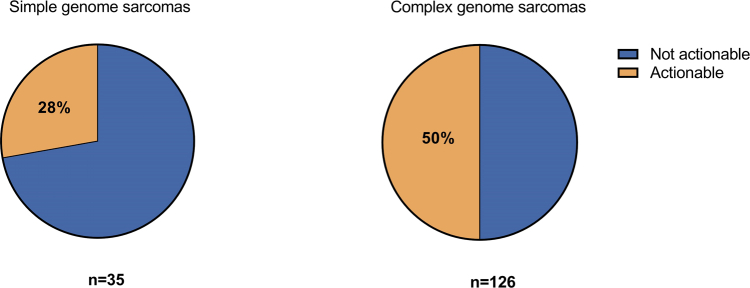


A total of 161 patient samples was successfully analyzed using WGS. Genomic alterations that were found to be clinically relevant, i.e. WGS-based alterations for which a therapy is available either as regular therapy or a clinical trial, were reported. Results are depicted in Figure 2. An actionable target was found for 74 patients (46%), multiple targets were detected by WGS for 35 patients (22%) and the WGS analysis did not lead to the discovery of therapeutic options for 87 patients (54%). The percentage of actionable targets found differed across STS subtypes, as can be seen from Supplementary Figure S1, available at https://doi.org/10.1016/j.esmoop.2025.105287. The percentage of actionability was highest for DDLPS (100%) and malignant peripheral nerve sheath tumor (MPNST, 100%), followed by angiosarcoma (63%) and UPS/sarcoma NOS (43%). No actionable targets were found for the five myxoid liposarcomas, and actionability for the 36 leiomyosarcomas was low [*n* = 7 (19%)]. Supplementary Figure S2, available at https://doi.org/10.1016/j.esmoop.2025.105287 shows actionable targets found in simple genome sarcomas and complex genome sarcomas. Actionable targets were found for 28% (10/ of 35) of simple genome sarcomas, versus 50% (63 of 126) for the complex genome sarcomas.

**Supplementary Figure S3:**
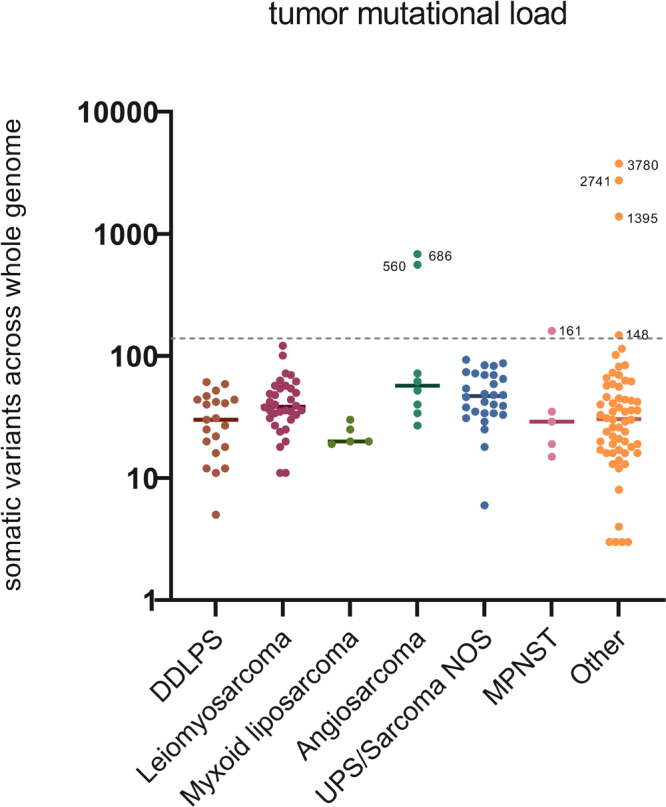


STS generally tend to have a low TML as shown in the current data (Supplementary Figure S3, available at https://doi.org/10.1016/j.esmoop.2025.105287). There are, however, notable cases in which a high TML (here proposed as >140 mutations/genome) was found: three pleomorphic dermal sarcomas (3780, 2741 and 1395 mutations/genome), two angiosarcomas (686 and 560 mutations/genome), one MPNST (161 mutations/genome) and one extraskeletal osteosarcoma (148 mutations/genome). The high mutational load in pleomorphic dermal sarcomas and angiosarcomas was UV-induced, as evidenced by an UV-signature in these samples.

**Figure 3:**
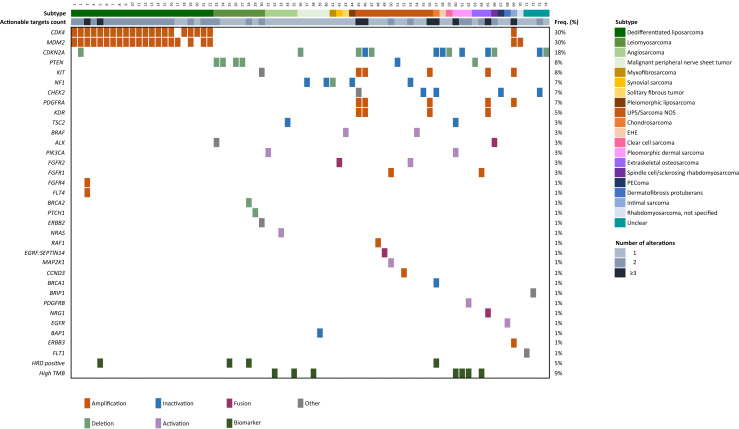
**Oncoplot showing actionable targets found by WGS per sample.** EHE, epithelioid hemangioendothelioma; HRD, homologous recombination deficiency; Freq., frequency; Sarcoma NOS, sarcoma not otherwise specified; PEComa, perivascular epithelioid cell tumor; TMB, tumor mutational burden; UPS, undifferentiated pleomorphic sarcoma.

Figure 3 gives an overview of actionable targets found by WGS analysis. As expected, actionable targets were found for all DDLPS samples represented by amplification of *CDK4* and/or *MDM2*, events that are known to frequently cooccur in DDLPS (*n* = 19). Next to this, deletion or loss-of-function of *CDKN2A* was commonly observed as a genomic event [*n* = 13 (18%)]. This, together with alterations in *CDK4* and *CCND3*, caused CDK4/6 inhibitors to be the most frequently suggested therapeutic option [*n* = 34 (47%)]. For a total of 22 patients (14%), the WGS result was followed by biomarker-driven therapy. This included the subtypes DDLPS, angiosarcoma, solitary fibrous tumor, UPS/sarcoma NOS, MPNST, extraskeletal osteosarcoma, rhabdomyosarcoma and perivascular epithelioid cell tumors. As these patients were enrolled in ongoing clinical trials, data on therapy duration and therapy response are not available. The main reasons to not start therapy based on actionable targets found at WGS (*n* = 51) were as follows: regular therapeutic options still available or no clinical necessity for systemic therapy yet (*n* = 17), very rapid disease progression or poor performance score (*n* = 10), patient was not eligible for study or withdrawal (*n* = 8), no benefit from therapy expected (*n* = 6) and other (*n* = 10).

## Discussion

We investigated whether WGS improves the guidance of individualized systemic therapy in STS. With these data, we aimed to identify patient subgroups that particularly benefit from WGS through the identification of new therapeutic options. Furthermore, we acquired data to determine the optimal time point to perform WGS during the disease course and gained insight into why WGS-informed therapy was not always administered when a systemic therapy option was found based on WGS results. Data from 161 patients that had successful WGS were analyzed. Actionable targets were found in 46% of patients, leading to WGS-informed therapy in 14% of cases. The highest rates of actionability were found for complex genomic sarcomas including DDLPS, angiosarcoma and UPS/sarcoma NOS subtypes. The major reasons to not start biomarker-driven medical therapy were no clinical necessity for WGS-informed therapy, rapid disease progression and/or poor performance status.

Several initiatives to investigate the clinical utility of genomic profiling exist. The European Society for Medical Oncology (ESMO) Precision Medicine Working Group published their recommendations for the use of next-generation sequencing (NGS) to inform therapy decisions in patients with advanced cancers.^21^ For advanced sarcoma, NGS is recommended for certain subtypes that might have a molecular target that is clinically actionable. These include *ALK* fusions in inflammatory myofibroblastic tumors, *COLIA1-PDGFB* fusions in dermatofibrosarcoma protuberans and *INI1/SMARCB1* mutations/deletions in epithelioid sarcoma. Previous studies on NGS in STS report rates of actionable alterations in 30% to 50% of cases across different sarcoma types, which is consistent with our study.^22^, ^23^, ^24^, ^25^ This wide range reflects different sequencing panel designs, variability in the definition of ‘actionable target’ and diversity of sarcoma types studied. Gounder et al. analyzed NGS data of 7494 STS and bone sarcomas representing 44 histological tumor types and observed large differences in actionability for genomically complex sarcomas versus translocation-associated sarcomas (36% versus 18%, respectively).^22^ Eventually 29% of this entire cohort was enrolled in a matched trial to receive therapy based on their genomic profile. The use of WGS to detect genomic alterations for the guidance of medical therapy is still at an early phase and has not yet been widely studied for STS. In the Zero Childhood Cancer Program, WGS was applied in 252 high-risk pediatric patients with cancer, including sarcoma, to identify potential therapeutic targets.^26^ In 40% of cases, WGS identified molecular alterations of which 71% were actionable targets. Of all patients for which an actionable target was reported, 43 (32%) received the recommended agents. Of the 62 pediatric sarcoma patients included, 9 (15%) eventually received targeted therapy based on this molecular profiling. In one sarcoma, a novel fusion was detected. Watkins et al. published the results of a study on WGS for diagnosis and management of STS.^27^ A total of 67 patient samples were analyzed using WGS. In 22 cases (33%), at least one potential target for therapy was identified by WGS. Five of them (12%) were *MDM2* amplifications that were already known through standard testing. In our study, *MDM2* amplification accounted for 30% of actionable targets found.

TML is generally low in sarcoma but can be used as biomarker to stratify patients who may benefit from immune checkpoint inhibitors therapy. It is hypothesized that a high number of mutations give rise to an increased generation of neoantigens, thereby enhancing the immunogenicity of tumors and making them more sensitive to immunotherapy. Although TML and TMB are often used interchangeably, TML refers to the total number of nonsynonymous somatic mutations across the genome. TMB generally also takes synonymous mutations into account; however, these mutations are less likely to be involved in tumor immunogenicity because they do not result in amino acid changes.^28^ In the current data, seven patients were found to have a high TML for which immune checkpoint inhibitors were suggested. In a cohort that enrolled 81 patients from the Dutch Drug Rediscovery Protocol study, the effect of pembrolizumab was tested based on a high TML across multiple cancer types, including three sarcomas, assessed by WGS analysis.^28^ This study showed clinical benefit (as defined as objective response or stable disease ≥16 weeks) in 21% of the patients in the cohort harboring a TML between 140-290, and 42% in the cohort with TML >290. In a study by Nacev et al., high TMB evaluated by NGS in STS was reported highest for UPS, angiosarcoma and uterine leiomyosarcoma.^29^ However, it is not yet known whether the cutoff that was used in this study (10 mutations per megabase) is the appropriate threshold for TMB as predictive biomarker in sarcoma to respond to immune checkpoint blockade. Further TMB studies in relation to therapy response in STS are needed to inform our understanding.

Molecular profiling for drug target identification is generally carried out by panel-based NGS. Rare cancers such as sarcoma may be disadvantaged by targeted panel sequencing, since coverage is based on mutations frequently found in common cancers that may not occur in rare cancers. Considering the heterogeneity of STS, the broad scope of WGS may be particularly impactful to uncover previously unknown genetic variants in both coding and noncoding regions.^9^ In addition to this, WGS is more likely to detect structural variants, a common event in sarcoma that often requires RNA analysis, compared with NGS and whole-exome sequencing.^8^^,^^13^^,^^27^^,^^30^ WGS is the most complete test as it can determine not only mutations, copy number variants, structural variants, HRD and microsatellite instability, but also oncogenic fusions, mutational signatures, pharmacogenetics and germline findings.^18^ This avoids the use of different tests for different tumors. Furthermore, previous studies of WGS data from longitudinally collected metastatic samples from patients undergoing systemic treatment have shown that actionable targets remain remarkably stable. This indicates that a single WGS analysis at time of metastasis is generally sufficient to provide essential information about biomarkers or treatment opportunities.^31^^,^^32^

Despite these obvious advantages, there are still obstacles for the implementation of WGS. WGS analysis provides large amounts of informative data, requiring costly storage and accurate handling of the data by experienced people. However, because of the comprehensive nature of WGS, these data are extremely suitable for future research and the raw data can be easily shared within the research community. This makes retrospective second use possible for the discovery and validation of new clinically relevant biomarkers. A single WGS test is far more expensive than NGS. The current costs for WGS in The Netherlands are about €2000, whereas NGS is half this price. However, costs for WGS will decrease. Not only will the cost of the test itself decrease but WGS is ideally suited for automation and developing a standardized protocol for laboratory work will also be cost saving. Next to this, compared to WGS, NGS panels still do not cover all relevant genomic changes and are often performed in a sequential manner, using time, costs and available tumor tissue which delays efficient clinical decision making. Our data show that a significant proportion of patients for whom an actionable alteration was revealed by WGS never received targeted therapy for various reasons.

When there is no clear clinical reason to perform WGS, it would be better to avoid using this method. Ideally, WGS is carried out at the beginning of the diagnostic workup to save doing other molecular tests, or when a clinical need or question arises during the disease course that could lead to access to therapies that are not standard treatment. Our study included 36 leiomyosarcomas but only a few cases had an actionable target. We also confirmed results of standard testing that *MDM2* and/or *CDK4* amplification was actionable in all 22 cases of DDLPS. This again implies that meaningful use of WGS for STS in clinical settings requires appropriate preselection of cases to avoid unnecessary costs.

Initiatives such as The 100 000 Genomes Project generate large amounts of valuable WGS data.^12^^,^^13^ The data used for the current study is an independent dataset derived from various studies, all of which involved samples that were sequenced and analyzed by Hartwig. Hartwig makes these data available to cancer research institutes and hospitals, and their data is used by research groups all over the world. Combining such data cohorts can amplify their impact and serve as a validation of findings from previous studies, which is crucial to build robust evidence in the specialized field of rare cancers. In addition to our results on the application of WGS to find targetable alterations that could support clinical decisions on systemic therapy, our study presents a real-world perspective on the clinical application of WGS by examining how WGS is actually integrated into the disease course in clinical practice. This is reflected in our analysis of the time points at which clinicians apply WGS during the disease course, offering insights into the use of WGS in a routine care setting. By exploring the reasons why WGS-guided therapeutic options were not followed, even when actionable findings were identified, we added important context to the discussion around implementation of WGS in clinical decision making. Our findings not only support previous findings but also provide insights aimed at improving the practical deployment of WGS in daily sarcoma care. We believe our approach significantly contributes to the understanding of how WGS is currently used in sarcoma and how it can be more effectively integrated into patient management.

There are some important limitations that need to be acknowledged. A limitation is the heterogeneity within the cohort. Our study comprised a variety of histological subtypes at different disease stages, with various pretreatments, and was therefore not powered to examine biomarker sub cohorts. Another limitation is the fact that the therapy that has been given and survival data could not be shared since all patients were enrolled in ongoing clinical trials. This limits the interpretation of the clinical utility of WGS for STS. Nonetheless, our data highlight the importance of WGS for specific STS cases. Complex karyotype sarcomas, particularly angiosarcoma and UPS/Sarcoma NOS are most likely to clinically benefit from WGS by giving access to tumor-agnostic therapies. Our study provides a basis for further, prospective studies on WGS in STS.

Effective targeted therapies for most STS are lacking. Their under-studied mutational landscape, complex biology and the absence of classically actionable drivers make it a challenging field for drug development. Although the role of clinical sequencing is still controversial in patients with sarcoma given the low mutational burden and lack of recurrent actionable targets, tumor-agnostic therapy by WGS is a major step forward in improving the number, and hopefully the efficacy of therapeutic options that could be offered for STS. This will increase the equity of access to therapies, broaden the spectrum of available therapies and contribute to the drug development process, potentially leading to faster approvals and earlier access for patients. Although not discussed in detail in this paper, implementation of WGS into the diagnostic process could help to refine STS diagnoses. Further studies should investigate WGS-driven analysis of STS subtypes and their molecular alterations with the aim to eventually develop functional therapies.

## Supplementary data

Supplementary Figure Caption
