# Supplementary material for: The role of whole-genome sequencing for guiding systemic therapy in patients with soft tissue sarcoma
Source: ESMO Open. 2025 Jun 11;10(6):105287. doi: 10.1016/j.esmoop.2025.105287 (PMC12192325; doi:10.1016/j.esmoop.2025.105287)
Supplement: Supplementary Figure Caption [file mmc1.docx]

**Supplementary Figure S1**. Percentage of samples with actionable alterations found by WGS across STS subtypes.

Abbreviations: DDLPS, dedifferentiated liposarcoma; MPNST: malignant peripheral nerve sheath tumor; Sarcoma NOS, sarcoma not otherwise specified; UPS, undifferentiated pleomorphic sarcoma.

**Supplementary Figure S2**. Percentage of samples with actionable alterations found by WGS across STS with simple and complex genomes.

**Supplementary Figure S3.** Tumor mutational load (somatic variants across whole genome) from WGS analysis across subtypes.

Abbreviations: DDLPS, dedifferentiated liposarcoma; Sarcoma NOS, sarcoma not otherwise specified; UPS, undifferentiated pleomorphic sarcoma. The dotted line separates tumors with low TML from high TML
